# An investigation into the potential for wind turbines to cause barotrauma in bats

**DOI:** 10.1371/journal.pone.0242485

**Published:** 2020-12-31

**Authors:** Michael Lawson, Dale Jenne, Robert Thresher, Daniel Houck, Jeffrey Wimsatt, Bethany Straw

**Affiliations:** 1 National Renewable Energy Laboratory, Golden, Colorado, United States of America; 2 Cornell University, Ithaca, New York, United States of America; 3 West Virginia University, Morgantown, West Virginia, United States of America; Tianjin University, CHINA

## Abstract

The high rates of bat mortality caused by operating wind turbines is a concern for wind energy and wildlife stakeholders. One theory that explains the mortality is that bats are not only killed by impact trauma, but also by barotrauma that results from exposure to the pressure variations caused by rotating turbine blades. To date, no published research has calculated the pressure changes that bats may be exposed to when flying near wind turbines and then used these data to estimate the likelihood that turbines cause barotrauma in bats. To address this shortcoming, we performed computational fluid dynamics simulations of a wind turbine and analytical calculations of blade-tip vortices to estimate the characteristics of the sudden pressure changes bats may experience when flying near a utility-scale wind turbine. Because there are no data available that characterize the pressure changes that cause barotrauma in bats, we compared our results to changes in pressure levels that cause barotrauma and mortality in other mammals of similar size. This comparison shows that the magnitude of the low-pressures bats experience when flying near wind turbines is approximately 8 times smaller than the pressure that causes mortality in rats, the smallest mammal for which data are available. The magnitude of the high-pressures that bats may experience are approximately 80 times smaller than the exposure level that causes 50% mortality in mice, which have a body mass similar to several bat species that are killed by wind turbines. Further, our results show that for a bat to experience the largest possible magnitude of low- and high-pressures, they must take very specific and improbable flight paths that skim the surface of the blades. Even a small change in the flight path results in the bat being hit by the blade or experiencing a much smaller pressure change. Accordingly, if bats have a physiological response to rapid low- and high-pressure exposure that is similar to other mammals, we conclude that it is unlikely that barotrauma is responsible for a significant number of turbine-related bat fatalities, and that impact trauma is the likely cause of the majority of wind-turbine-related bat fatalities.

## Introduction

Over the last two decades, there has been a dramatic increase in the deployment of wind turbines. Between 2002 and 2019 in the United States, the installed capacity of wind turbines increased from 2.5 GW to over 105 GW [1], and at the end of 2019 there were over 60,000 [2] utility-scale turbines installed across the country, accounting for more than 6.3% of the nation’s electricity generation [3]. Projections suggest that 20% of the U.S. electricity production may come from wind by 2030 [4]. As the number of deployed turbines grows, it is increasingly important to mitigate negative environmental impacts from wind turbine deployment.

One adverse outcome is the large number of bat deaths that occur at some wind turbine installations [5–11]. It was initially assumed that impact trauma was responsible for the overwhelming majority of turbine-related bat deaths, and even though bats have remarkable echolocation and aerobatic abilities, the speed at which turbine blades move make it difficult for bats to detect and avoid impact with the moving blades. However, one highly cited 2008 study [12] found bat carcasses near wind turbines that appeared to have died from internal hemorrhaging that is characteristic of barotrauma (trauma that results from exposure to sudden changes in ambient pressure), with no characteristic signs of impact trauma, such as broken bones. This finding has led several researchers to hypothesize that the pressure fluctuations caused by rotating turbine blades are large enough to cause barotrauma, and that simply flying sufficiently close to wind turbine blades may cause mortality in bats.

**Fig 1:**
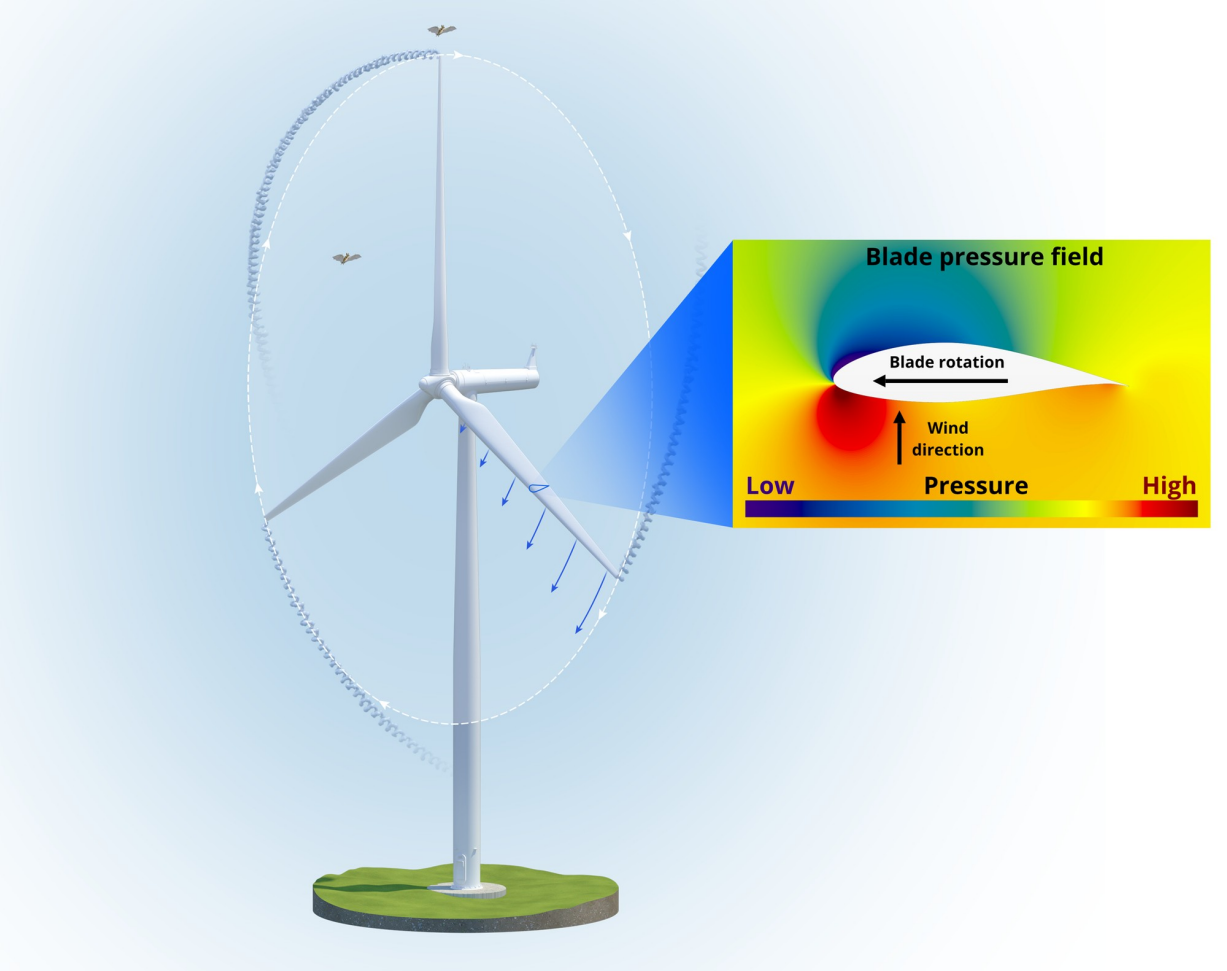
Pressure changes caused by an operating wind turbine. A low-pressure region forms over the blade suction surface (downwind side) and a high-pressure region forms over the blade pressure surface (upwind side of the blade) as a result of local flow accelerations. A region of low pressure is also created by the vortex that forms as air flows around the tip from the blade pressure side to the suction side. The tip-vortex propagates downstream in the direction of the wind as shown.

**Fig 2:**
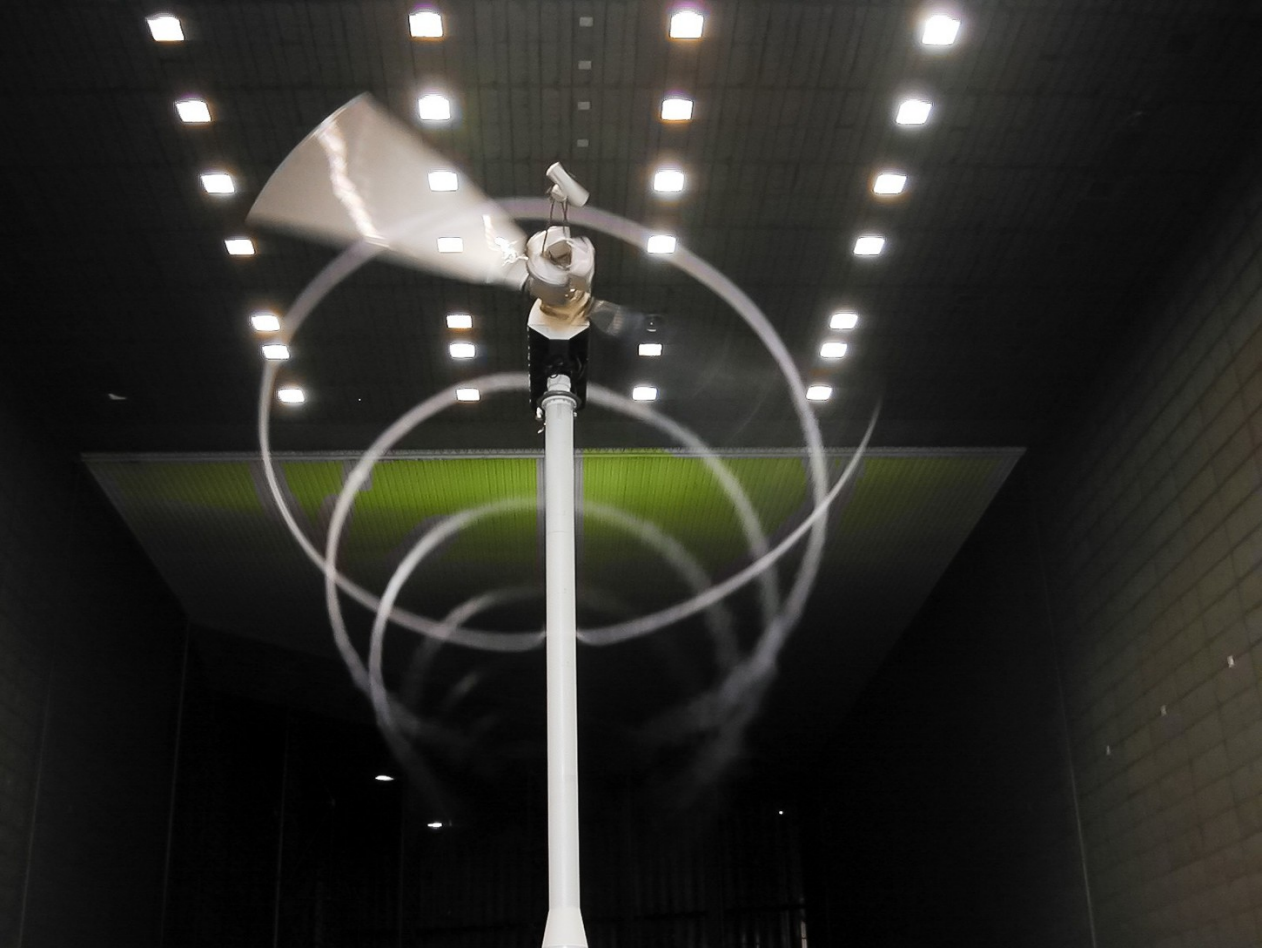
Smoke visualization of a blade-tip vortex at the national aeronautics and space administration’s ames research center 40-by-80-ft wind tunnel. The visible helical vortex indicates the region of low pressure caused by the blade-tip vortex. Note that this visualization was performed under low-turbulence conditions in a wind tunnel, allowing the vortex to advect downwind with the mean flow with its helical structure intact. In the atmosphere, where the turbulence intensity is typically much higher, the vortex structure is commonly unrecognizable within one rotor diameter downstream of the rotor plane. Photo by Lee Jay Fingersh, National Renewable Energy Laboratory, 55062.

A depiction of the pressure changes caused by an operating wind turbine that could potentially cause barotrauma are presented in Figs 1 and 2. There are high- and low-pressure regions that form over the blade surfaces because of flow accelerations and a low-pressure region that forms in the blade-tip vortex [13]. These pressure changes decay approximately as the inverse square of the distance from the blade surface and the center of the vortex. Accordingly, for a bat to experience a large-magnitude high- or low-pressure that could cause barotrauma its flight path must skim the blade surface or the center of the vortex, as described in detail in later sections.

The magnitude of the pressure changes caused by the turbine blade and blade-tip vortex increase approximately with the square of the relative blade speed, and the blade speed increases approximately linearly with wind speed over the range of wind speeds that bats typically fly [13]. Accordingly, the potential for a turbine to cause barotrauma increases rapidly with wind speed. It is also important to note that while blade-tip vortices can persist for several rotor diameters downwind, their strength and associated pressure field quickly decay as viscosity and turbulence cause the vortices to stretch and loose their coherent structure [14]. Thus, the potential for blade-tip vortices to cause barotrauma decreases as the vortices propagate downstream from the turbine.

In 2008, Baerwald et al. [12] presented the first evidence supporting the hypothesis that bats are killed by barotrauma around wind turbines. They collected 188 bats killed at a wind farm and found that 87 (46%) did not show signs of external injuries consistent with impact trauma. Seventy-five of the dead bats were necropsied. Of the 75, 32 (42%) had obvious external injuries and 43 (57%) showed signs of internal hemorrhaging of the thoracic cavity and/or the abdominal cavity, injuries that are characteristic of barotrauma, but exhibited no external injuries. Baerwald et al. [12] concluded that barotrauma was responsible for the bat deaths where no impact trauma was evident and also concluded that barotrauma is likely a significant cause of wind-turbine-related bat fatalities. Baerwald et al. [12] note that bats may be particularly susceptible to barotrauma because they have a thinner blood-gas barrier than terrestrial mammals. Many avian species also have a thin blood-gas barrier, but have other anatomical adaptations, such as a rigid lung structure and exceptionally strong capillaries, that may make birds less susceptible to barotrauma. Indeed, no published research has shown evidence that wind turbines cause barotrauma in avian species.

In contrast, a 2012 forensic study of bats killed at wind farms by Rollins et al. [15] concluded that traumatic injury resulting from blade impact is the most significant cause of mortality, with barotrauma being at most a minor factor. Rollins et al. [15] noted that the forensic indications that have been commonly used to identify barotrauma in bats found around wind turbines may be unreliable because of the rapid postmortem onset of edema unrelated to barotrauma [15, 16]. More recently, preliminary biomechanical [17] and aerodynamic [18] studies have attempted to determine if the pressure variations bats experience when flying near wind turbines have the potential to cause barotrauma, but these studies have been generally inconclusive.

Although bat mortality from impact trauma has been directly observed using thermal video recordings [6, 7], the body of scientific research has not conclusively demonstrated that wind turbines cause barotrauma in bats that results in mortality. Nevertheless, barotrauma is widely cited in both scientific literature (e.g., [19]) and by the media (e.g., [20–22]) as a significant cause of bat deaths that occur at wind farms. Accordingly, our objective in this work is to provide quantitative data that can be used to estimate the likelihood that wind turbines cause barotrauma. To accomplish this, we calculate the pressure changes caused by a representative utility-scale wind turbine using computational and analytical methods and then compare them to pressure levels that are likely to cause barotrauma in bats. Because there are no data available on pressure change levels that cause barotrauma in bats, we used the available mortality threshold data from several mammals to estimate the likelihood that wind turbines cause barotrauma in bats.

The following section of this paper describes the analytical and computational methods we used to estimate the pressures that bats could experience flying near the blade surface and the tip vortex of a utility-scale wind turbine. Next, in the *Results and Discussion* section, we present the results and identify the range of low- and high-pressure changes that bats are likely to experience when flying near utility-scale wind turbines. In the *Results and Discussion*, we also estimate the likelihood that wind turbines could cause fatal barotrauma by comparing our results to changes in pressure levels that cause barotrauma and mortality in other mammals of similar size. Finally, in the *Conclusion* section, we review the implications of our findings and discuss how the results can help the conservation community, regulatory agencies, and the wind energy industry engage in better-informed discussions and prioritize research that could help minimize bat fatalities caused by wind turbines.

## Methods

### Turbine selection

In order to estimate the pressure fluctuations that bats experience when flying near a utility-scale wind turbine, we selected a representative turbine geometry and operating conditions. We chose the National Renewable Energy Laboratory (NREL) 5 MW reference turbine because it is open source, widely accepted by the wind energy community as a standard for research studies, and it has a three-bladed, variable-speed, variable-pitch design and operating characteristics that are similar to the vast majority of utility-scale wind turbines operating today. Table 1 presents the characteristics of the NREL 5-MW reference turbine and further details are presented by Jonkman et al. [23]. It is important to note that because the design and operating characteristics of the vast majority of utility-scale wind turbines are similar, the results developed with the NREL 5 MW reference turbine that are presented herein are generally extendable to today’s utility-scale wind turbine fleet and to the next generation of land-based and offshore wind turbines currently under development.

**Table 1:** Design and operating characteristics of the NREL 5-MW reference turbine [23].

Rating	5 MW
Rotor orientation, Configuration	Upwind, Three blades
Control system	Variable speed, Collective pitch
Rotor, Hub diameter	126 m, 3 m
Hub height	90 m
Cut-in, Rated, Cut-out wind speed	3 m/s, 11.4 m/s, 25 m/s
Cut-in, rated rotor speed	6.9 rpm, 12.1 rpm
Rated tip speed	80 m/s

It is important to note that there are ways that turbine size may influence bat interactions with turbines that are not considered herein. The next generation of offshore wind turbines will have rotors of 200 m diameter or more [24] and the size of land based turbines is also rapidly increasing [1]. Because rotor RPM decreases linearly with turbine radius to maintain an optimal ratio of blade-tip speed to wind speed (i.e., tip-speed ratio, [13]), how bats interact with large turbines will influence the risk of collision with the rotating blades. Specifically, if bats fly mostly near the nacelle, where the blades of large turbines move relatively slow, the number of fatalities per MW of installed wind capacity may be reduced for farms comprised of large turbines. Conversely, if bats have a tendency to fly in areas away from the hub where blade speed is high the opposite may be true. Consideration of these and other similar questions are outside the scope of the present study and are left for future work.

### Identification of relevant wind speeds

In 2005, Arnett et al. [7] found that bat fatalities around wind turbines decrease with increasing wind speed. This has consistently been supported by subsequent fatality monitoring studies [8, 10, 11, 25]. Further, numerous curtailment studies show that there is a significant decrease in bat fatalities when the wind turbine cut-in speeds (i.e., the wind speed where the turbine first starts operating) are increased [9, 16, 26], strongly suggesting that bat activity around wind turbines decreases with increasing wind speed. Cryan et al. [27] used thermal cameras to study bat activity around individual turbines and did not record any bat activity when wind speeds were over 9.6 m/s, and over 90% of bat activity was recorded below wind speeds of 7.1 m/s. Similarly, Wellig et al. [28] found that 95% of bat activity in an area of the European Alps occurred when wind speeds were below 4.4 m/s.

The magnitude of the pressure fluctuations caused by an operating turbine increase rapidly with oncoming wind speed. Specifically, rotor rotational speed and blade velocity increase with oncoming flow speed to maintain optimal turbine performance, whereas the magnitude of the pressure field increases approximately with the square of the blade speed relative to the wind (see [13] for a detailed discussion of the aerodynamics of wind turbines). Thus, the low- and high-pressure regions that bats might be exposed to while flying near an operating wind turbine increase rapidly with oncoming wind speed.

Based on this information, we chose to consider wind speeds of 5 m/s, 7.5 m/s, and 10 m/s to study the potential for wind turbines to cause barotrauma. The 5 m/s and 7.5 m/s speeds represent conditions in which bats commonly fly around wind turbines, whereas the 10 m/s case represents a wind speed on the high end of the wind speed range wherein bats are rarely observed to fly [27–29]. Because the magnitude of pressure fluctuations caused by the turbine increases quickly with wind speed, the 10 m/s scenario will produce significantly larger pressure fluctuations than the 5 m/s and 7.5 m/s scenarios, and is the wind condition wherein bats would experience the largest pressure fluctuations when flying around turbines. Table 2 presents the operating characteristics of the NREL 5 MW reference turbine for the wind speeds considered.

**Table 2:** NREL 5 MW turbine operating conditions considered.

Wind Speed, *u*_∞_ (m/s)	Blade pitch angle (deg)	Tip-speed ratio	Relative wind speed at 90% blade span (m/s)	Angle of attack at 90% blade span (deg)
5	0.0	10.0	45.3	7.2
7.5	0.0	7.7	52.5	9.1
10	0.0	7.6	68.7	9.2

### Calculation of the blade pressure field

In calculating the blade pressure field, our objective was to estimate the largest pressure variations that bats could be exposed to when flying near an operating wind turbine blade. Accordingly, instead of simulating the pressure field around the entire blade, we chose to simplify our simulations and only calculate the pressure field at the 90% span location (i.e., 90% of the distance from the turbine hub to the blade tip). At this span location, the blade velocity is 90% of its maximum, which occurs at the blade tip, whereas three-dimensional flow effects that occur near the tip and decrease the magnitude of the blade pressure field are not yet significant [13]. Thus, bats will experience the largest pressure variations when flying close to the 90% blade span location.

We performed simulations by solving the Reynolds-averaged Navier-Stokes (RANS) equations of turbulent fluid flow using the commercial computational fluid dynamics (CFD) software STAR-CCM+ [30]. We used the k-*ω* SST (shear stress transport) RANS model [31] to capture the effects of turbulence as this model has been used successfully in several wind turbine CFD studies [32–35]. In particular, many of these studies demonstrated good agreement with experimental measurements under flow conditions similar to those considered in this work, as previously described by Simms et al. [36].

**Fig 3:**
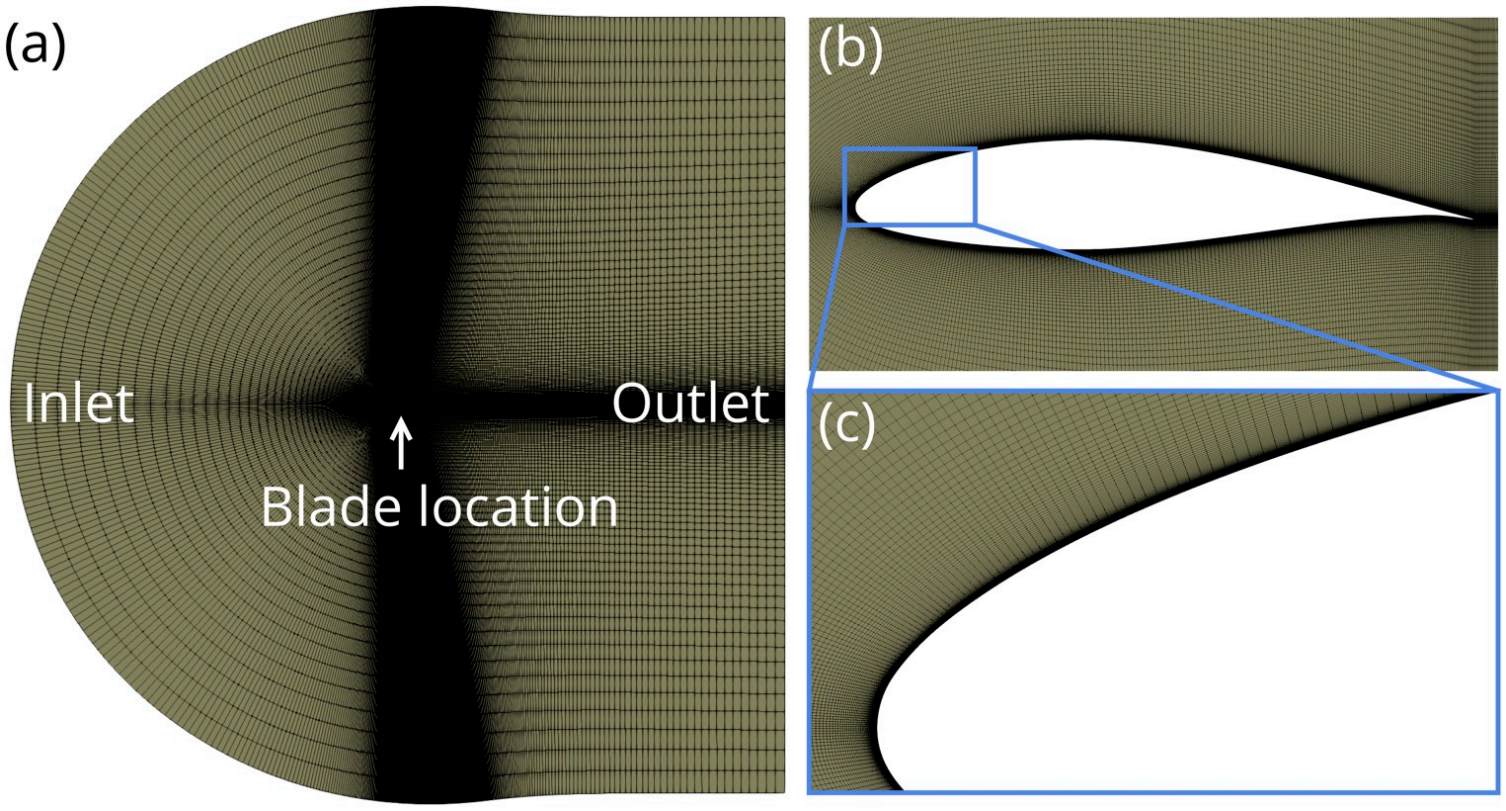
Computational mesh, which includes (a) the c-grid computational domain, (b) the near-blade mesh topology, and (c) the boundary layer mesh extrusion near the blade surface.

Because the flow at the 90% span location is approximately two-dimensional for the conditions considered [33], we simplified the CFD simulations by performing two-dimensional simulations of the blade cross section at this spanwise location. At the 90% span location, the NREL 5 MW reference turbine blade chord length is 2.31 m and the profile is a National Advisory Committee for Aeronautics (NACA) 64-series airfoil with an airfoil thickness of 17% of the blade chord length [23]. We used Pointwise [37] to generate a standard c-grid structured hexahedral grid shown in Fig 3. We extruded a boundary layer mesh from the blade surface and the y+ value of the first cell off the blade was kept below 1 for all simulations, ensuring the turbulent boundary layer was appropriately resolved. A spatially uniform velocity profile with a turbulence intensity of 5% was specified for the inlet boundary condition and a pressure outlet boundary condition was applied across the back surface of the computational domain, as illustrated in Fig 3.

To ensure the accuracy of our CFD simulations, we performed a grid convergence study and validated our numerical methods against canonical experimental and numerical data. For the grid convergence study, we created three grids comprising 131,000, 263,000, and 526,000 elements and compared the pressure field calculated using each grid. The results of the grid convergence study show that increasing the number of grid elements beyond 263,000 resulted in a <1% change in the magnitude of the pressure field; therefore, the 263,000 element grid was used for all simulations.

**Fig 4:**
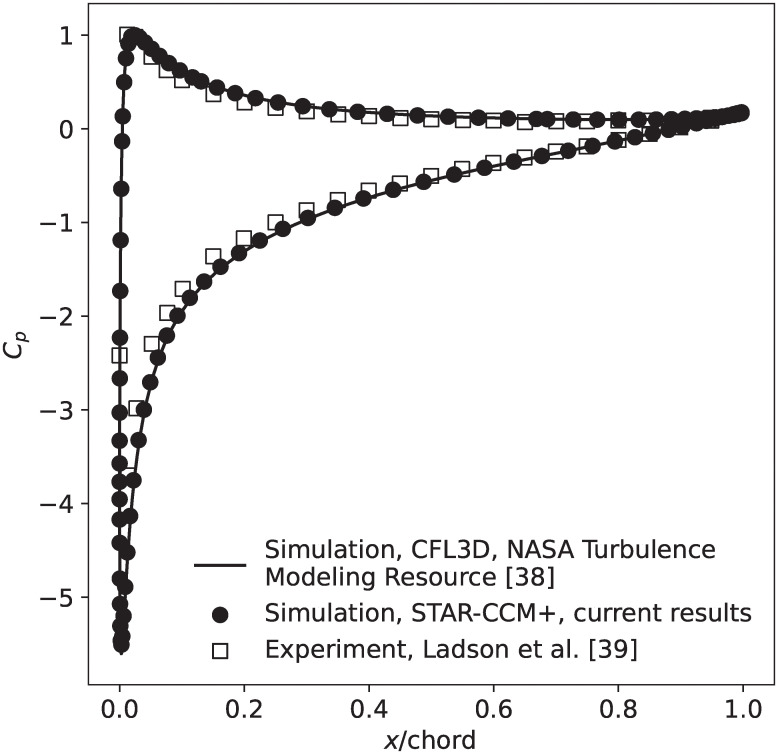
Comparison of the blade surface pressure coefficient, *C*_*p*_, for a NACA 0012 airfoil predicted in the current study using STAR-CCM+ compared with experimental results from Ladson et al. [39] and CFD results generated using CFL3D and a 897-by-257 computational grid [38]. All results are shown at a blade chord Reynolds number of 6 million.

We validated the CFD simulation methodology by using STAR-CCM+ to calculate the pressure field created by a NACA 0012 airfoil at a 10°angle of attack and a Reynolds number of 6 million, and comparing the results to numerical and experimental data from the NASA Turbulence Modeling Resource [38]. For these simulations, we used the computational mesh provided by the National Aeronautics and Space Administration (NASA) Turbulence Modeling Resource [38] that has a number of grid cells and a mesh topology nearly identical to that presented in Fig 3. Specifically, we compared the pressure coefficient,

$$\begin{array}{c} C_{p}=\frac{p-p_{\infty }}{\frac{1}{2}\rho_{\infty }u_{rel}^{2}} \end{array}$$ (1)

over the blade surface with experimental data from Ladson et al. [39] and simulation results from CFL3D [40] provided by the NASA Turbulence Modeling Resource [38]. In Eq 1, *p* is the blade surface pressure, *p*_∞_ is the atmospheric pressure, *ρ*_∞_ is the density of air, and *u*_*rel*_ is the wind speed relative to the blade. Overall, our predictions of the pressure coefficient, which is representative of the pressure distribution over the blade surface, show excellent agreement with both the experimental and numerical data, as shown in Fig 4. In order to correctly predict the pressure coefficient, simulations must also accurately model the pressure field surrounding the blade, thus the results presented in Fig 4 provide confidence that the STAR-CCM+ simulation methodology used for this work accurately predicts the pressures bats could be exposed to around an operating wind turbine.

### Calculation of the blade-tip vortex pressure field

Blade-tip vortices form as air from the pressure side of the blade flows around the blade tip, creating a rotational flow structure that propagates downstream with the mean flow (see Fig 1). CFD predictions of tip vortices are highly dependent on the mesh resolution and the turbulence modeling method and are still an active area of research (e.g., [41]) that is beyond the scope of this study. We therefore chose to use an analytical vortex model developed by Vatistas et al. [42] to predict the velocity and pressure field created by the NREL 5 MW reference turbine,

$$\begin{array}{c} v_{\theta }\left(\bar{r}\right)=\frac{\Gamma_{vortex}}{2\pi r_{c}}\frac{\bar{r}}{\sqrt{1+{\bar{r}}^{4}}} \end{array}$$ (2)

*v*_*θ*_(*r*) is the tangential velocity within the vortex, Γ_*vortex*_ is the vortex strength, and $\bar{r}$ is the radial distance from the vortex center, *r*, normalized by the vortex core radius, *r*_*c*_.

The pressure within the vortex, *p*_*vortex*_, is related to the rotational velocity (i.e., tangential velocity) of the vortex, *v*_*θ*_, through the radial component of the inviscid Navier-Stokes equations,

$$\begin{array}{c} \frac{dp_{vortex}}{dr}=\rho \frac{v_{\theta }^{2}}{r} \end{array}$$ (3)

where *ρ* is the density of air. Following the methodology described by Moriarty [43], we substituted Eq 2 into Eq 3 and integrating yields an equation relating pressure inside the vortex as a function of distance from the vortex center. If we further assume that the pressure far away from the vortex (i.e., where *r* → ∞) is the atmospheric pressure, *p*_∞_, we get the following result,

$$\begin{array}{c} p{\left(r\right)}_{vortex}-p_{\infty }=\frac{\rho \Gamma {}_{vortex}^{2}}{8\pi^{2}r_{c}^{2}}[tan^{-1}(\frac{r^{2}}{r_{c}^{2}})-\frac{\pi }{2}] \end{array}$$ (4)

In order to use Eq 4 to determine the pressure field, we needed to estimate the tip vortex strength and the core radius.

**Fig 5:**
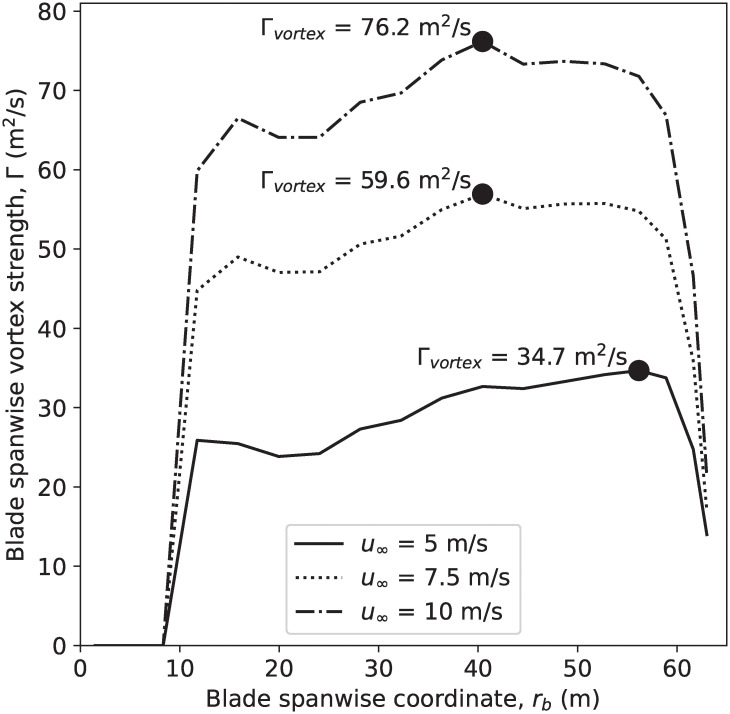
Blade vorticity distribution along the blade of the NREL 5-MW reference turbine calculated using OpenFAST. Note that solid circles identify the maximum spanwise vorticity, which corresponds to the strength of the corresponding blade-tip vortex.

We are able to estimate Γ_*vortex*_ using a combination of potential flow theory and the OpenFAST wind turbine modeling code [44]. From potential theory, we know that the strength of the blade-tip vortex is equal to the maximum bound vorticity strength along the blade span, where *r*_*b*_ is the coordinate along the blade span. We are therefore able to calculate the blade-tip vortex strength for each wind speed considered by first calculating the bound vorticity strength along the blade span and then taking the maximum value. We know that the bound vorticity along the blade is related to the blade lift distribution, *L*(*r*_*b*_), by the Kutta-Joukowski theorem,

$$\begin{array}{c} L\left(r_{b}\right)=\rho v\left(r_{b}\right)\Gamma \left(r_{b}\right)dr_{b} \end{array}$$ (5)

where *v*(*r*_*b*_) is the relative air velocity at each radial blade location. Using OpenFAST, we can calculate both lift distribution and the local velocity along the NREL 5 MW reference turbine blade. Substituting this information into Eq 5, we solved for bound vorticity distribution and the strength of the blade-tip vortex and the result is shown in Fig 5.

We estimated the core radius of the blade-tip vortex using a correlation developed by Martin et al. [45, 46] based on a set of experimental measurements of tip vortices. Martin et al. [45, 46] measurements indicate that $r_{c}=0.05\bar{c}$, where $\bar{c}$ is the average blade chord length. For the NREL 5 MW reference turbine, *r*_*c*_ = 0.17, and using this information we are able to evaluate Eqs 2 and 4 to determine the velocity and pressure field caused by the blade-tip vortex.

## Results and discussion

### Pressure bats experience flying near the blade surface

**Fig 6:**
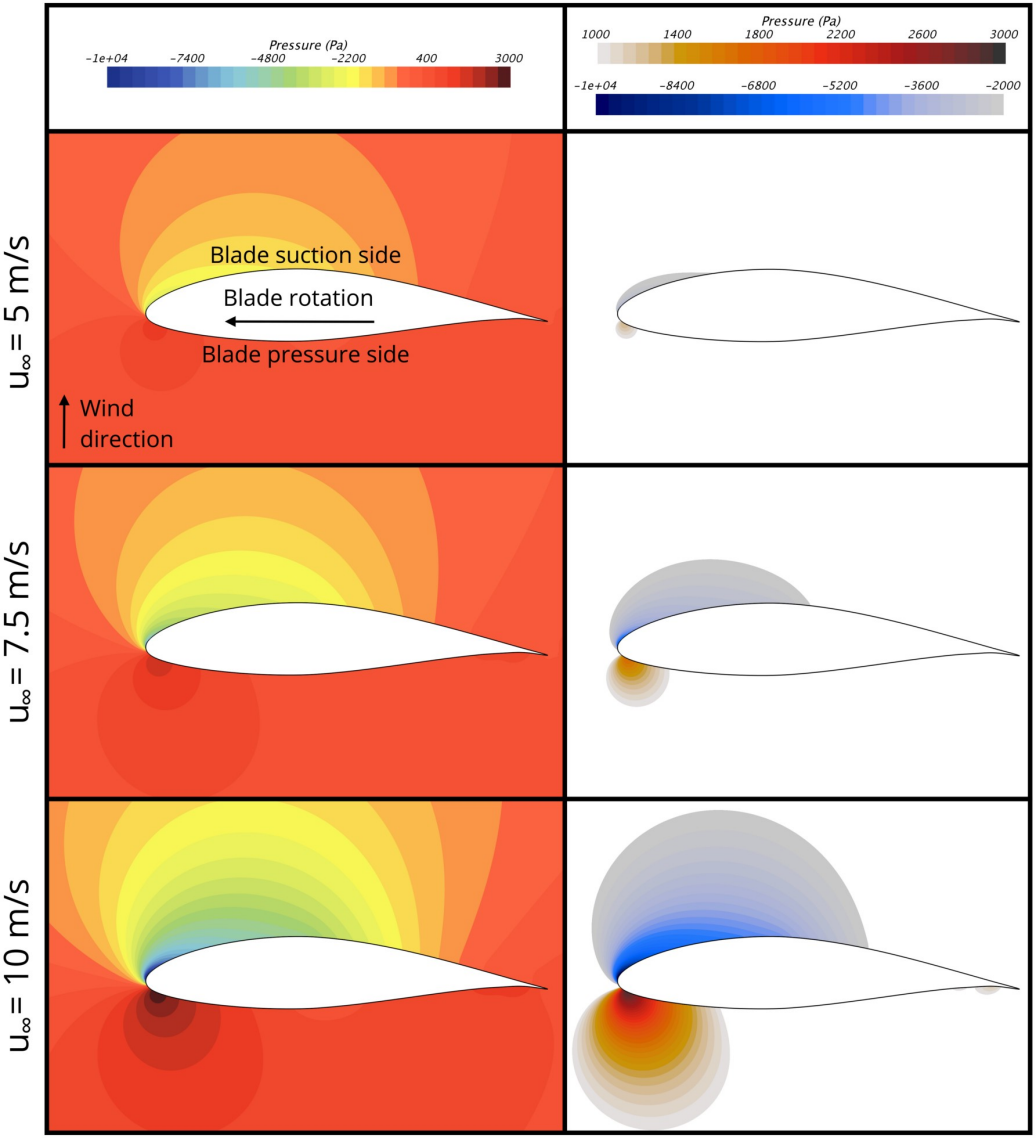
Blade surface pressure for wind speeds of 5 m/s, 7.5 m/s, and 10 m/s. (Left frames) Full-field pressure contours. (Right frames) Pressure contours showing regions of the pressure field where the pressure is lower than -2000 Pa and higher than 1000 Pa, with respect to atmospheric pressure.

Fig 6 presents the pressure fields that develop around the wind turbine blade with respect to atmospheric pressure. The full field contours in the left frames show the entire field and clearly illustrate the low- and high-pressure regions that form on the suction side (i.e., downwind side) and pressure side (i.e., upwind side) of the blade, respectively. To better visualize the extent of the low- and high-pressure regions, the right frames of Fig 6 show pressure contours where the pressure is below -2000 Pa and above 1000 Pa, which corresponds to regions that are approximately 2% below and 1% above atmospheric pressure, respectively. Fig 6 clearly shows that the low- and high-pressure regions are very localized near the leading edge of the blade and the pressure quickly decays toward atmospheric pressure with increasing distance from the blade surface.

In order to estimate possible flight paths and the pressures bats could experience when flying through the blade pressure field, we used a Lagrangian particle tracking method [30]. We modeled bats as Lagrangian particles, with a mass of 25 g, a density of 1000 kg/m^3^, a wing area of 0.0117 m^2^, and an associated lift coefficient of 1. These properties were chosen to approximate the characteristics of a Hoary bat (*Lasiurus cinereus*), one of the species most commonly found at wind energy facilities. Significant variations in any proprieties of the Lagrangian particles that represented the bats had negligible effect on the flight paths because the aerodynamic forces acting on the Lagrangian particles were insignificant compared to the particle inertia over the time it takes for the particles to pass by the blade. This result indicates that the possible flight paths close to wind turbine blades will be the same for the majority of bat species, regardless of their body mass or other relevant anatomical characteristics, such as wing area. We calculated the possible bat flight paths abound the NREL 5 MW reference turbine blade when bats are flying at speeds of 0 m/s, ±5 m/s, and ±10 m/s, with respect to the wind. By considering these flight conditions, we covered the range of flight velocities at which bats typically fly [47–49]. Further, by considering these flight speeds and directions, we capture potential flight paths for bats approaching the rotor from both the upwind and downwind directions and flight paths of bats flying in the rotor plane.

**Fig 7:**
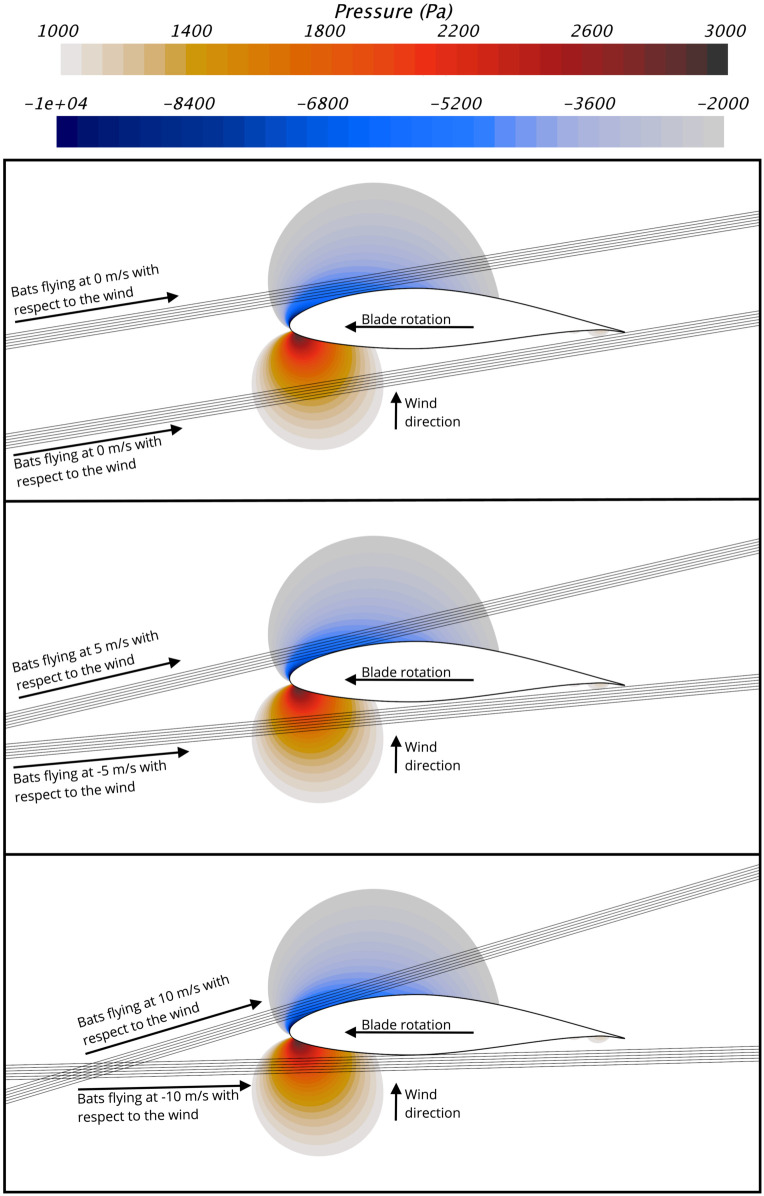
Bat flight paths by the 90% blade span location of the NREL 5 MW wind turbine operating at a wind speed of 10 m/s. (Top) Flight paths for bats flying at 0 m/s with respect to the wind. (Middle) Flight paths for bats flying at ±5 m/s, with respect to the wind. (Bottom) Flight paths for bats flying at ±10 m/s, with respect to the wind. The pressure contours show the pressure variations caused by the blade that are below -2000 Pa and above 1000 Pa, with respect to atmospheric pressure. For scale, note that the bat flight paths are separated by 20 mm in the direction normal to the flight paths.

**Fig 8:**
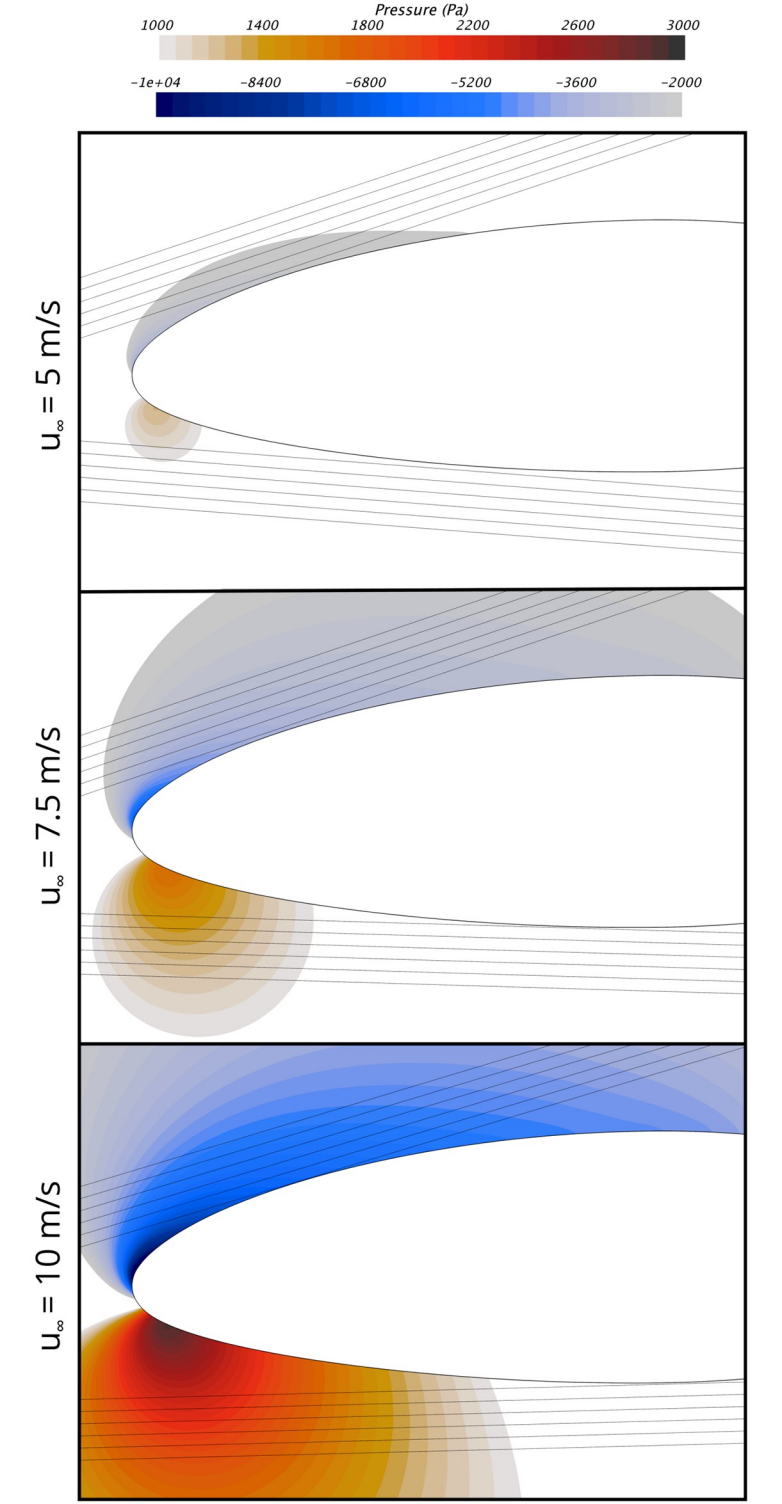
Bat flight paths by the 90% blade span location of the NREL 5 MW reference turbine from the upwind and downwind direction when the turbine is operating at wind speeds of 5 m/s, 7.5 m/s, and 10 m/s. Note that only the front section of the blade is shown and that the flight paths are for a bat flight speed of 10 m/s, with respect to the oncoming wind direction. For scale, note that the bat flight paths are separated by 20 mm in the direction normal to the flight paths.

Fig 7 shows the simulated bat flight paths for the wind speed of 10 m/s. As shown, we modeled six flight paths through the low-pressure region and six through the high-pressure region for each flight condition considered. The starting location of the simulated flight paths was set so the closest flight path passed <1 mm from the blade surface, and so the farthest flight path was approximately 100 mm away from the blade. The results in Fig 7 show that physical constraints resulting from the fact that the blade is moving much faster than bats fly limit the regions of the blade pressure field bats can experience without being struck by the blade. It can be observed that the faster bats fly, the closer to the low- and high-pressure peaks they can pass without being struck by the blade. Therefore, we focus on the the 10 m/s flight speed from here on. Fig 8 shows the bat flight paths near the leading edge of the blade for each wind velocity considered at the 10 m/s bat flight speed. Because the blade speed increases significantly with wind speed (see Table 2), the bat flight paths are different for each wind speed. It is clear from Fig 8 that even when flying at the 10 m/s flight speed, bats cannot encounter the largest-magnitude low and high pressures caused by the blade. It follows that it is not the largest pressure drop caused by the turbine blade that is relevant for estimating the potential for wind turbines to cause barotrauma, but rather the pressure that it is physically possible for a bat to encounter.

**Fig 9:**
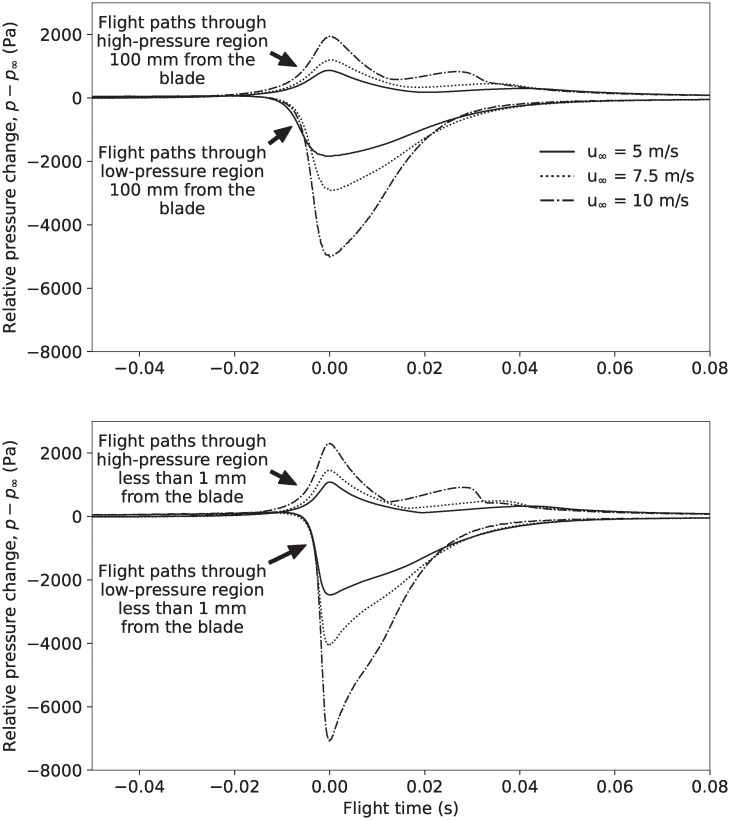
Pressure vs. time history for bat flight paths near the NREL 5 MW reference wind turbine blade at the 90% blade span location for wind speeds of 5 m/s, 7.5 m/s, and 10 m/s. (Top) flight paths 100 mm from the blade surface and (bottom) flight paths <1 mm from the blade surface. Note that the pressure-time histories have been shifted to the minimum or maximum pressure that occurs at 0 s. Time before 0 seconds is when bats are approaching the peak low or high pressure, and time after 0 seconds is when bats are flying away from peak low or high pressure.

To better quantify the pressure bats experience while flying along each flight path, we plotted the pressure versus time history of the flight paths, as shown in Fig 9. We clearly see that the magnitude of the low-pressure peak experienced is significantly higher than the magnitude of the high-pressure peak. Further, regardless of the flow speed, bats are exposed to elevated pressure levels for very short durations. It takes approximately 0.02 seconds for the pressure to change from atmospheric pressure (i.e., represented by 0 Pa herein) to the pressure at the low- and high-pressure peaks. It then takes less than 0.08 seconds for the pressure to recover to within 5% of the atmospheric pressure, again, regardless of the flow speed. Thus, from the perspective of a bat flying near a wind turbine blade, the entire duration of time where the pressure is more than 5% higher or lower than the atmospheric pressure is less than 0.1 seconds. Finally, for clarity, we have summarized the minimum and maximum pressures bats can experience when flying near the NREL 5 MW wind turbine at the 90% blade span location in Table 3.

**Table 3:** The minimum and maximum pressures bats could experience when flying near the 90% blade span location at a flight speed of 10 m/s and near the blade-tip vortex of the NREL 5 MW reference turbine, with respect to atmospheric pressure.

Wind speed, *u*_∞_ (m/s)	Minimum pressure along flight path through low-pressure region (Pa)		Minimum pressure in the blade-tip vortex (Pa)	Maximum pressure along flight path through high-pressure Region (Pa)	
	<1 mm from the blade	100 mm from surface		<1 mm from the blade	100 mm from surface
5	-2477	-1837	-944	1086	873
7.5	-4057	-2916	-2545	1462	1198
10	-7077	-5011	-4556	2304	1947

### Pressure bats experience flying near the blade-tip vortex

**Fig 10:**
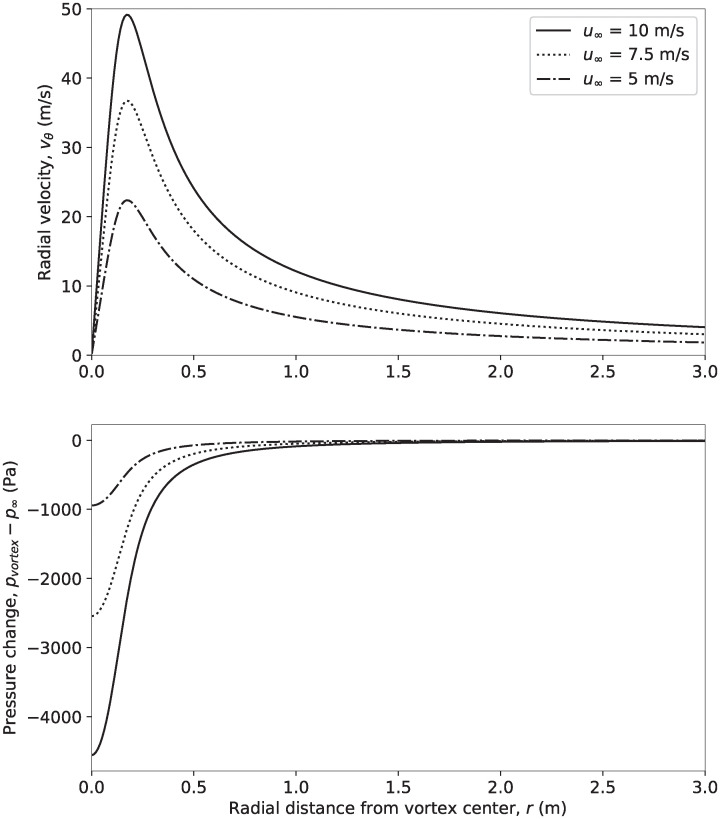
(Top) Velocity caused by the blade-tip vortex of the NREL 5 MW reference turbine calculated using Eq 2. (Bottom) Pressure change caused by the tip-vortex calculated using Eq 4.

We used the Vatistas vortex model previously described to calculate the velocity and pressure changes caused by the blade-tip vortex of the NREL 5 MW wind turbine and the results are presented in Fig 10. The model predicts that the minimum pressure occurs at the center of the vortex, where the rotational velocity of the vortex is 0, and then increases back toward the atmospheric pressure with increasing distance from the vortex center. Overall, the minimum pressure caused by the blade-tip vortex is less than the pressure a bat would experience flying 100 mm from the blade surface at the 90% span location, as shown in Table 3.

Although, Fig 10 shows that the blade-tip vortex causes a significant region of low pressure, there are at least two factors that limit the likelihood that blade-tip vortices cause barotrauma. First, the vortices cause a high velocity approximately 1 meter from the vortex core, as shown in Fig 10. The effect of this high velocity is to push any bat that approaches the vortex away from the vortex center, potentially limiting the low-pressure peak that a bat could experience because of the tip vortex. The second is that the physics of vortex formation suggest that the minimum pressure peak presented in Fig 10 and Table 3 is an overestimate of what a bat will actually experience because turbulence and viscous effects that tend to decrease vortex strength were not considered, as described in in the *Methods* section.

This occurs because the methodology used to estimate the vortex properties does not account for two important effects of viscosity. First, we assumed the bound circulation on the blade is instantaneously rolled up into the vortex when it forms. In reality, the shed vorticity will not be entrained into the vortex until it has propagated a certain distance downstream, resulting in a lower vortex strength and a smaller magnitude low-pressure peak due to diffusion caused by viscosity. Thus, our methodology inherently overestimates the initial vortex 0-m/strength. Second, viscosity causes the vortex core to increase in size over time as it propagates downstream away from the blade tip, rapidly decreasing the low-pressure peak of the vortex pressure field. For example, if the size of the viscous core increases by just 5% of the average blade chord length, the maximum pressure drop caused by the vortex at the 1s wind speed would decrease by approximately 75%. Although a detailed investigation of these effects is far beyond the scope of this work, we strongly suggest that the Vatistas vortex model provides a conservative estimate of the pressure bats would be exposed to in the tip vortex of the NREL 5 MW reference turbine.

### Comparison with data from other animals

**Fig 11:**
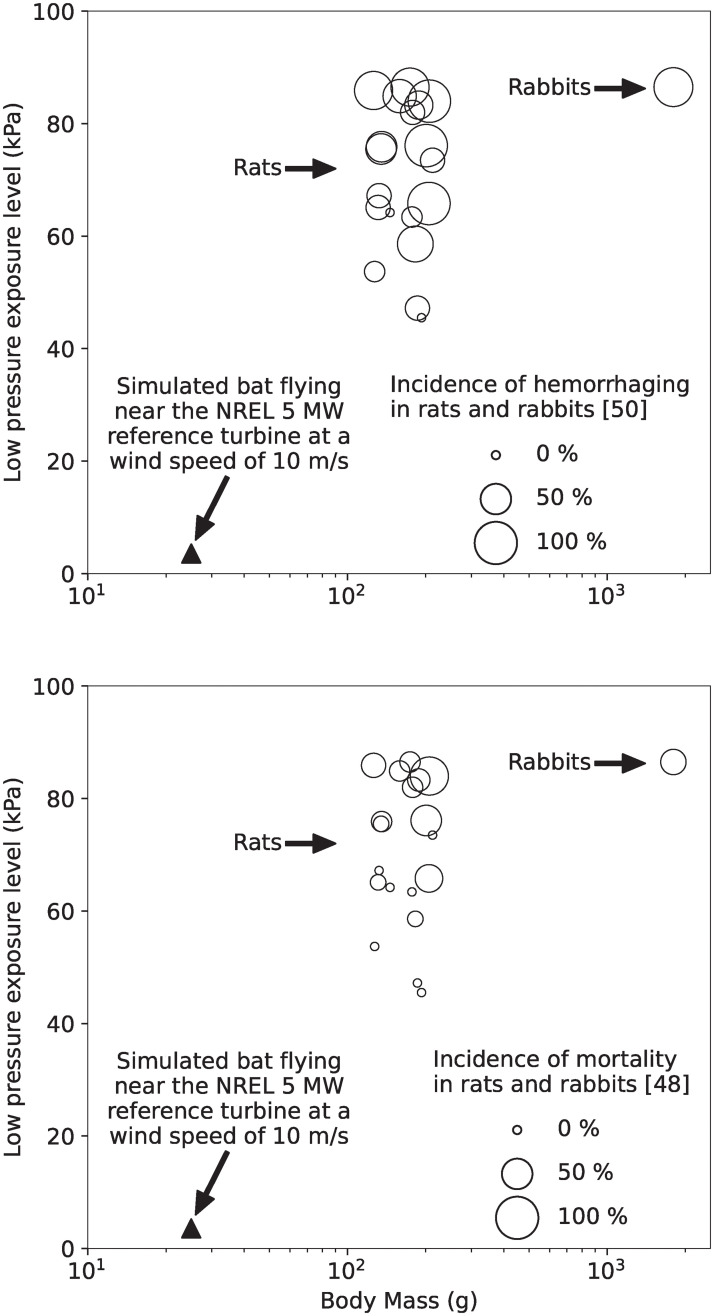
Occurrence of lung hemorrhaging (top) and mortality (bottom) in rats and rabbits as a result of sudden exposure to low pressures. The circles present the data from Junkui et al. [50] for rats and rabbits. The triangle indicates the largest magnitude low pressure our results indicate bats could be exposed to when flying near the NREL 5 MW wind turbine when the wind speed is 10 m/s. Note that there is no mortality or hemorrhaging data available for bats rapidly exposed to low pressures.

To estimate the likelihood that pressure changes caused by utility-scale wind turbines cause barotrauma, information describing the pressure levels that are harmful to bats is required. However, to date, no research has been performed to determine the effect that sudden exposure to low and high pressures has on bats. A review of the literature shows that Junkui et al. [50] have performed the only study investigating how rapid decompression affects the mortality of small mammals. In their 1996 study, Junkui et al. [50] exposed rats and rabbits to rapid decompression using a vacuum chamber. The rats and rabbits were anesthetized and placed in a chamber at 98 kPa (approximately 1 standard atmosphere). They were then exposed to rapid decreases in pressure between -45.5 kPa and -86.5 kPa for durations between 0.0021 s and 1.9 s (compared to an approximately 0.1 s pressure exposure duration for bats flying near wind turbines), and incidence of lung hemorrhaging and mortality from barotrauma were recorded. It is relevant to note that the duration of the pressure changes considered by Junkui et al. [50] are of similar duration to the pressure changes that bats experience when flying near a utility-scale wind turbine, as previously discussed. Fig 11 presents the results of Junkui et al. [50], showing the percentage of lung hemorrhaging and mortality that was observed, plotted as a function of body mass and pressure exposure level. It can be observed that no mortality and low incidence of lung hemorrhaging were observed when the rats were exposed to low-pressure levels less than 58 kPa below atmospheric pressure. For reference, the largest magnitude low pressure we predicted that bats are exposed to when flying near the NREL 5 MW wind turbine is 7 kPa, and is plotted as a solid triangle in Fig 11.

The rats studied by Junkui et al. [50] have an average body mass of 168 g; almost an order of magnitude larger than smaller bats that are known to be killed by wind turbines. Further, because we only have data describing how rats and rabbits respond to low-pressure exposure, and have only a single data point for rabbits, we are unable to develop an allometric scaling curve that lets us relate lung hemorrhaging and mortality to low-pressure exposure as a function of body mass. Still, from Fig 11, it can be observed that the lowest pressure we estimate bats are exposed to around wind turbines when the wind speed is 10 m/s (7077 Pa below atmospheric pressure) is over 8 times lower than 58600 Pa below the atmospheric pressure that is needed to cause a single instance of mortality in rats. If we further consider wind speeds of 7.5 m/s and 5 m/s, wherein bats are much more likely to be found flying near turbines, the pressures we estimate bats are exposed to (4057 Pa and 2477 Pa below atmospheric pressure) are 14 and 24 times lower, respectively, than the pressure needed to cause mortality in the rats.

**Fig 12:**
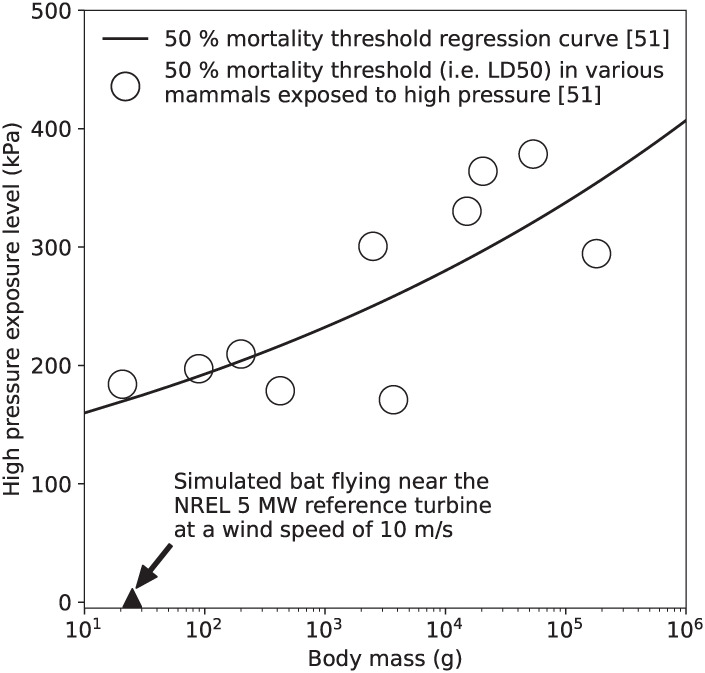
50% mortality threshold levels for various mammals rapidly exposed to high pressures from Richmond et al. [51]. The triangle indicates the largest magnitude high pressure we predict bats could be exposed to when flying near the NREL 5 MW wind turbine when the wind speed is 10 m/s. Note that there is no mortality data available for bats rapidly exposed to high pressures.

In contrast, there is a significant amount of published research that describes how rapid exposure to high pressures affects mammals (e.g., [51, 52]). Although the majority of research in this area was performed in the 1950s and 1960s to study the biological effects that blast waves from explosions have on mammals, results from Richmond et al. [51] can be used here to make some general conclusions regarding the potential for wind turbines to cause barotrauma in bats. Fig 12 presents data compiled by Richmond et al. [51] on the high pressure exposure level that causes mortality in 50% of the animals tested (i.e. the LD50). It is notable that mice have an average body mass of 20.7 g, similar to hoary bats, and have an LD50 high pressure exposure of 184000 Pa with respect to atmospheric pressure, or more than 80 times higher than the highest pressure bats are exposed to around a wind turbine at a wind speed of 10 m/s. Fig 12 also presents an LD50 regression curve developed by Richmond et al. [51]. If bats response to sudden exposure to high pressures is similar to that of other mammals, the regression analysis suggests that the LD50 level for bats is 73 times higher than the highest pressure bats experience at a wind speed of 10 m/s.

We believe that the methods we used to estimate the pressures that bats could be exposed to when flying near wind turbines are conservative for four reasons:

1. We estimated the pressure variations that bats experience when flying around an operating wind turbine when the wind speed is 10 m/s. At this wind speed, the pressure variations caused by the turbine are significantly larger than they are at wind speeds below 7.5 m/s, at which bats more commonly fly [27, 28], and below which most turbine-related bat fatalities occur [9, 17, 26].
2. The largest-magnitude low and high pressures bats can experience when flying near the blade occur when bats pass <1 mm from the blade, as shown in Fig 8 and Table 3. For a bat’s mouth and nose to pass <1 mm from the surface of the blade, exposing air-filled internal organs to the largest-magnitude pressure variations, it is almost certain that part of a bat’s body would be struck by the blade, causing impact trauma and potentially death. It therefore seems likely that the pressure variations bats experience without being struck by the blade are closer to the pressures that exist 100 mm (i.e., they likely have a much smaller magnitude) from the blade surface than the pressures that are <1 mm from the surface (see Fig 9 and Table 3).
3. We calculated the blade pressure at the 90% blade span location because this is the location of the blade that causes the largest pressure variations, as described in the Methods section, and sections of the blade closer to the tip or hub will cause smaller pressure variations. In effect, there is a very small region where the largest-magnitude low or high pressures can be experienced, and the pressure variations over the vast majority of the rotor disk will be significantly lower than what is reported herein for the 90% span location.
4. As detailed in Methods section, the simplified analysis methodology used to estimate the pressures near the blade-tip vortex likely results in a significant overestimate of the magnitude of the pressure variation.

## Conclusion

Impact trauma and pulmonary barotrauma are widely cited as the proximate causes of bat fatalities around wind facilities [53]. Although impact trauma has been clearly shown to cause bat deaths around turbines (e.g., through infrared video recordings [27]), the evidence supporting the barotrauma hypothesis comes primarily from a single field study [12] that documented evidence of pulmonary edema, and no indication of impact trauma, in dead bats found in close proximity to wind turbines. A more recent forensic study of bats killed by wind turbines [15] found that impact trauma was responsible for the majority of the deaths and questions the methods that have typically been used to diagnose barotrauma in deceased bats, disputing the hypothesis that barotrauma is responsible for a significant number of wind-turbine-related bat fatalities.

In this article, we investigated the potential that utility-scale wind turbines cause barotrauma by using computational and analytical fluid dynamics methods. Specifically, we calculated the pressure changes bats could experience flying near wind turbines and compared the results to pressure mortality threshold data for mammals of similar size and weight because there are no data that describe the physiological response of bats to rapid changes in pressure. The results show that the magnitude of the lowest pressure bats could be exposed to when flying in proximity to an operating wind turbine is 8 times smaller than the pressure that causes mortality in rats. Rats have an average body mass of 186 g and are the mammal closest in body mass to bats for which data are currently available (see Fig 11). Similarly, the magnitude of the highest pressures bats are likely exposed to when flying in proximity to a wind turbine are more than 80 times below the LD50 exposure level for mice, which have a body mass that is similar to many bats that are killed by wind turbines (see Fig 12). Further, we found that the low- and high-pressure regions generated by the blade are localized to a small region near the leading edge of the blade and decay rapidly with increasing distance from the blade. Bats must therefore take a very specific and unlikely flight path to enter the regions of low- and high-pressure caused by the turbine blade without being struck, as shown in Figs 7 and 8. We also estimated the magnitude of the low-pressure region caused by the tip vortex and found that it was 13 times smaller magnitude of low-pressure exposure that causes mortality in mice. We therefore conclude that if bats have a physiological response to rapid low- and high-pressure exposure that is similar to other mammals it is unlikely barotrauma is responsible for a significant number of turbine-related bat fatalities.

Baerwald et al. [12] suggested that bats may be more susceptible to barotrauma than other mammals because they have a particularly thin blood-gas barrier, and bats may have other flight adaptations that make them more vulnerable to barotrauma than other mammals. Still, even if the pressure changes close to the blade are large enough to cause barotrauma, the region where barotrauma could occur will be small compared to the area swept by the blade (see Figs 6 and 8). Thus, the probability of a bat being struck by the blade will be significantly higher than the probability of a bat passing close enough to the blade to experience barotrauma. It has also been suggested that bats may experience sublethal barotrauma, such as damage to the tympanic membrane or other internal injuries, that allow them to fly far away from turbines before they succumb to their injuries [15, 54, 55]. If this is the case, further study is required so that mortality estimators [56, 57] that are used to determine the number of fatalities caused by renewable energy facilities can account for such circumstances. Ultimately, to fully understand the potential for wind turbines to cause both lethal and sublethal barotrauma, data describing how bats respond to sudden changes in pressure is needed.

Regardless of whether collisions with turbine blades or barotrauma is the primary cause of bat fatalities at wind turbines, the solutions to the problem are the same—develop effective deterrent technologies, and smart curtailment strategies (i.e., shutting down turbines during the periods of highest risk) to minimize bat fatalities. We believe the results presented herein will help the conservation community, regulatory agencies, and the wind energy industry engage in better-informed discussions on the interactions between bats and wind turbines and prioritize research needs to help achieve this goal.
